# DNA repair pathways underlie a common genetic mechanism modulating onset in polyglutamine diseases

**DOI:** 10.1002/ana.24656

**Published:** 2016-05-06

**Authors:** Conceição Bettencourt, Davina Hensman‐Moss, Michael Flower, Sarah Wiethoff, Alexis Brice, Cyril Goizet, Giovanni Stevanin, Georgios Koutsis, Georgia Karadima, Marios Panas, Petra Yescas‐Gómez, Lizbeth Esmeralda García‐Velázquez, María Elisa Alonso‐Vilatela, Manuela Lima, Mafalda Raposo, Bryan Traynor, Mary Sweeney, Nicholas Wood, Paola Giunti, The SPATAX Network, Alexandra Durr, Peter Holmans, Henry Houlden, Sarah J. Tabrizi, Lesley Jones

**Affiliations:** ^1^ Department of Molecular Neuroscience, Institute of NeurologyUniversity College LondonLondon WC1N 3BGUnited Kingdom; ^2^ Department of Clinical and Experimental Epilepsy, Institute of NeurologyUniversity College LondonLondon WC1N 3BGUnited Kingdom; ^3^ Department of Neurodegenerative Disease, Institute of NeurologyUniversity College LondonLondon WC1N 3BGUnited Kingdom; ^4^ Center for Neurology and Hertie Institute for Clinical Brain ResearchEberhard‐Karls‐UniversityTübingenGermany; ^5^ Inserm U 1127, CNRS UMR 7225, Sorbonne UniversitésUPMC University Paris 06 UMR S 1127, Institut du Cerveau et de la Moelle épinière (ICM)ParisFrance; ^6^ APHP, Department of GeneticsUniversity Hospital Pitié‐Salpêtrière75013 ParisFrance; ^7^ Université Bordeaux, Laboratoire Maladies Rares: Génétique et MétabolismeINSERM1211BordeauxFrance; ^8^ CHU Pellegrin, Service de Génétique Médicale, F‐33000BordeauxFrance; ^9^ Ecole Pratique des Hautes Etudes75014 ParisFrance; ^10^ Neurogenetics Unit, 1st Department of NeurologyUniversity of Athens Medical School, Eginition Hospital115 28 AthensGreece; ^11^ Neurogenetics Department, National Institute of Neurology and Neurosurgery“Manuel Velasco Suárez”Mexico City CP14269Mexico; ^12^ Department of BiologyUniversity of the Azores9500‐321 Ponta DelgadaPortugal; ^13^ Instituto de Investigação e Inovação em SaúdeUniversidade do Porto4150‐180 PortoPortugal; ^14^ Institute for Molecular and Cell Biology (IBMC)University of Porto4150‐180 PortoPortugal; ^15^ Laboratory of Neurogenetics, National Institute of AgingNIHBethesdaMD 20892, USA; ^16^ Neurogenetics Unit, National Hospital for Neurology and NeurosurgeryUniversity College London HospitalsLondon WC1N 3BGUnited Kingdom; ^17^ Ataxia Center, Institute of NeurologyUniversity College LondonLondon WC1N 3BGUnited Kingdom; ^18^ MRC Centre for Neuropsychiatric Genetics and Genomics, Institute of Psychological Medicine and Clinical NeurosciencesCardiff UniversityCardiffCF24 4HQUnited Kingdom

## Abstract

**Objective:**

The polyglutamine diseases, including Huntington's disease (HD) and multiple spinocerebellar ataxias (SCAs), are among the commonest hereditary neurodegenerative diseases. They are caused by expanded CAG tracts, encoding glutamine, in different genes. Longer CAG repeat tracts are associated with earlier ages at onset, but this does not account for all of the difference, and the existence of additional genetic modifying factors has been suggested in these diseases. A recent genome‐wide association study (GWAS) in HD found association between age at onset and genetic variants in DNA repair pathways, and we therefore tested whether the modifying effects of variants in DNA repair genes have wider effects in the polyglutamine diseases.

**Methods:**

We assembled an independent cohort of 1,462 subjects with HD and polyglutamine SCAs, and genotyped single‐nucleotide polymorphisms (SNPs) selected from the most significant hits in the HD study.

**Results:**

In the analysis of DNA repair genes as a group, we found the most significant association with age at onset when grouping all polyglutamine diseases (HD+SCAs; *p* = 1.43 × 10^–5^). In individual SNP analysis, we found significant associations for rs3512 in *FAN1* with HD+SCAs (*p* = 1.52 × 10^–5^) and all SCAs (*p* = 2.22 × 10^–4^) and rs1805323 in *PMS2* with HD+SCAs (*p* = 3.14 × 10^–5^), all in the same direction as in the HD GWAS.

**Interpretation:**

We show that DNA repair genes significantly modify age at onset in HD and SCAs, suggesting a common pathogenic mechanism, which could operate through the observed somatic expansion of repeats that can be modulated by genetic manipulation of DNA repair in disease models. This offers novel therapeutic opportunities in multiple diseases. Ann Neurol 2016;79:983–990

Over 30 human diseases are caused by expansion of unstable microsatellite sequences.^1^ Nine contain repeats that encode glutamine, usually referred to as the polyglutamine diseases (Table 1), and have common features, including autosomal‐dominant inheritance (except X‐linked spinal and bulbar muscular atrophy), genetic anticipation, neuronal involvement, and intracellular inclusions containing the cognate polyglutamine protein. The phenotypes vary, potentially reflecting differences in the temporal and regional expression and protein context of the disease‐causing expansions^2^ (see Table 1). There are currently no disease‐modifying treatments for these devastating conditions.

**Table 1:** Characteristics of the Polyglutamine Diseases

Repeat Disorder	Gene	Prevalence	Variance in AAO Explained by Repeat Length	Normal Range	Pathogenic Range	Somatic Instability
HD	*HTT*	3–10	50–60% (40–60%)	6–35	40–121	Yes
SCA1	*ATXN1*	0.16	64–76% (no detected heritable component)	6–38	45–83	Yes
SCA2	*ATXN2*	0.2	50–80% (17–59%)	15–31	33–500	Yes
SCA3	*ATXN3*	0.4	45–80% (46%)	12–44	52–87	Yes
SCA6	*CACNA1A*	0.04	26–52% (no detected heritable component)	4–18	20–33	Unknown
SCA7	*ATXN7*	0.12	71–88% (no detected heritable component)	3–19	37–460	Yes
SCA17	*TBP*	<0.02	Unknown	25–40	49–66	Unknown
DRPLA	*ATN1*	0.005–0.04	50–68%	6–35	48–93	Yes
SBMA	*AR*	0.65–2.00	29%	9–34	38–72	Yes

Epidemiology and CAG repeat ranges of polyglutamine diseases. Prevalence is given/100,000 European population. AAO = age at onset; HD = Huntington's disease; SCA = spinocerebellar ataxia; DRPLA = dentatorubral‐pallidoluysian atrophy; SBMA = spinal and bulbar muscular atrophy.

In the polyglutamine diseases, longer CAG repeat tracts lead to earlier age at onset (AAO), though the relationship varies between diseases (see Table 1).^3^, ^4^ Not all of the difference in AAO is accounted for by CAG repeat length, and in Huntington's disease (HD)^4^ and at least spinocerebellar ataxia (SCA) types 2 and 3,^5^ a substantial portion of this residual variance is heritable, suggesting the existence of additional modifying factors within the genome. The Genetic Modifiers of Huntington's Disease (GeM‐HD) genome‐wide association study (GWAS)^6^ found two genome‐wide loci associated with age at motor onset in HD on chromosomes 15 and 8, with two independent signals at the same locus on chromosome 15. A few SCA genetic modifiers have been proposed^3^, ^5^, ^7^, ^8^, ^9^ and no GWAS have been reported.

Genetic anticipation in these diseases occurs because the repeats are meiotically unstable and tend to expand over successive generations; most also show tissue‐specific somatic instability^10^ (see Table 1). In HD, somatic instability is expansion‐biased and age‐ dependent, with larger tracts more susceptible to expansion.^11^, ^12^ It occurs in postmitotic neurons and is prominent in striatum and cortex, tissues particularly affected in HD.^13^ Somatic instability has been linked to disease onset and progression in both human^14^ and mouse HD studies,^15^ and decreasing somatic expansion in HD model mice delays phenotype progression.^16^ Many of the principles of somatic instability in HD extend to SCAs.^1^, ^10^ Somatic instability^12^, ^17^, ^18^ has been attributed to the actions of DNA repair proteins, and as well as the individually associated variants, the GeM‐HD GWAS found significant association between age at motor onset and several DNA repair pathways.^6^ These GeM‐HD GWAS findings, along with evidence for somatic instability in other polyglutamine diseases (see Table 1), led us to hypothesize that variants in DNA repair genes might modify AAO in all polyglutamine diseases.

In this report, we demonstrate significant associations between variants in genes involved in DNA repair pathways and the AAO of polyglutamine diseases as a group as well as with some polyglutamine diseases individually.

## Patients and Methods

### Patients

Subject cohorts were gathered from the Neurogenetics Unit and Ataxia Center of the National Hospital for Neurology and Neurosurgery (London, UK), TRACK‐HD (Europe),^19^ SPATAX network (France), the University of Athens Medical School/Eginition Hospital (Athens, Greece), the National Institute of Neurology and Neurosurgery, Manuel Velasco Suarez (Mexico), and the University of Azores (Ponta Delgada, Portugal; Table 2). All subjects with polyglutamine diseases seen at any of the collaborators sites and willing to participate in research were enrolled regardless of their CAG repeat size or AAO. All studies were approved by local ethics committees, and all subjects gave written informed consent. For this study, we gathered samples and data for HD and SCAs 1, 2, 3, 6, 7, and 17; very few dentatorubral‐pallidoluysian atrophy (DRPLA) and spinal and bulbar muscular atrophy (SBMA) samples were available to us, so these diseases were not included. AAO and CAG repeat size was available for 1,462 patients (see Table 2). Given the varied phenotypes of polyglutamine diseases, motor onset (HD) or onset of the first progressive symptom as reported by the patient was used to determine AAO throughout all cohorts. Given the small number of patients, SCA17 was only considered in the combined SCA analysis.

**Table 2:** Cohort Characteristics

Cohort	Disease							
	HD	SCA1	SCA2	SCA3	SCA6	SCA7	SCA17	Total
Athens, Greece	351	0	0	0	0	0	0	351
Azores, Portugal	0	0	0	91	0	0	0	91
London, UK	0	30	66	45	69	7	1	218
Mexico	0	0	113	0	0	66	6	185
SPATAX, France	0	147	115	261	0	0	0	523
TRACK‐HD, Europe	94	0	0	0	0	0	0	94
Total	445	177	294	397	69	73	7	1,462
% M	49.4	54.2	48.6^a^	52.6	60.9	56.2	85.7	51.8^a^
Mean AAO ± SD (range)	45 ± 12.1 (6–82)	37 ± 10.5 (16–65)	33 ± 12.9 (8–73)	39 ± 11.6 (9–74)	57 ± 10.5 (18–76)	35 ± 17.6 (5–84)	30 ± 13.4 (8–44)	
Mean (CAG)n length ± SD (range)	44 ± 5.0 (37–92)	48 ± 5.3 (39–66)	42 ± 4.5 (33–64)	71 ± 4.4 (50–82)	22 ± 0.9 (21–26)	48 ± 11.1 (36–100)	51 ± 6.4 (42–58)	

a One subject had no sex information.

HD = Huntington's disease; SCA = spinocerebellar ataxia; % M = percentage of males; AAO = age at onset; SD = standard deviation.

### Single‐Nucleotide Polymorphism Selection Criteria and Genotyping

Single‐nucleotide polymorphisms (SNPs) were selected from the most significant genes (gene‐wide, *p* < 0.1) in the “DNA repair pathway cluster” from the GeM‐HD analysis (listed in Table S4 of the GeM‐HD article).^6^ SNPs from *RRM2B* and *UBR5* were added to this list because they are both members of GO:6281 “DNA Repair” (which, although nominally significant in GeM, did not reach *q* < 0.05 and was therefore not used to create the pathway cluster), both lie within a genome‐wide significant association peak in GeM‐HD, and both have significant gene‐wide *p* values (see Table S5 of the GeM‐HD article).^6^ For each gene, the most significant SNP was selected, along with a small number of proxy SNPs in close LD (*r* ^2^ > 0.8) with the most significant SNP that also showed association in GeM‐HD. Where possible, these proxy SNPs were chosen to have functional annotation (http://browser.1000genomes.org/index.html: accessed 12/6/14). If a gene contained two independent significant signals in GeM‐HD (e.g., *FAN1*), then the lead SNP for the second signal was included. Note that this selection procedure is not intended to give comprehensive coverage of the genes in question, but instead to highlight SNPs likely to be disease relevant. To guard against the effects of population stratification, SNPs were removed from the analysis if they had a Hardy‐Weinberg *p* value <0.001 in the whole data set. These procedures yielded 22 genotyped SNPs with success rates ranging from 94.2% to 98%, as described in Supplementary Table 1.

SNP genotyping was performed using custom KASP assays at LGC Genomics (Hertfordshire, UK). Gene‐level sense sequences were used to design SNP assays (see Supplementary Table 2). The assays for several SNPs were designed in reverse orientation to the chromosome (rs4150407, rs1805323, rs1037700, rs1037699, rs3512, and rs20579). For this reason, for all SNPs in reverse orientation to the chromosome (rs4150407, rs1805323, rs1037700, rs1037699, rs3512, and rs20579), genotypes resulting from these KASP assays will be complementary to those using HGVS nomenclature. This is reflected in Supplementary Table 3, where the minor allele for these SNPs differs from GeM‐HD,^6^ but corresponds to the same allele.

### Statistical Analyses

AAOs for the various diseases were corrected for repeat length using a similar method to the GeM‐HD GWAS.^6^ A linear regression was performed for each disease separately of ln(AAO) on expanded repeat length. Regression parameters are given in Table 3. These parameters were used to construct an expected value of AAO for each individual, based on their repeat length, which was subtracted from their actual AAO to give a residual. Association of each SNP with AAO was tested by performing a linear regression of these residuals on the number of minor alleles in the genotype in PLINK.^20^ The effect of gender on AAO (after accounting for CAG length) was also tested. Since this was nonsignificant for all disorders (results not shown), gender was not included in the calculation of residuals.

**Table 3:** Effects of Repeat Length of the Expanded Allele on the Age at Onset

Disease	Sample N	A	B	*p*
HD	445	6.119939	−0.052966	<2e‐16
SCA1	177	5.682974	−0.043694	<2e‐16
SCA2	294	5.799343	−0.056682	<2e‐16
SCA3	397	7.137211	−0.049477	<2e‐16
SCA6	69	5.96740	−0.08686	0.00268
SCA7	73	4.643231	−0.026023	2.94e‐5
SCA17	7	2.38659	0.01716	0.70

Results of fitting a linear regression ln(AAO) = A + B*(CAG)n. *p* value refers to the significance of the regression parameter (B) indexing the effect of repeat length.

HD = Huntington's disease; SCA = spinocerebellar ataxia.

The primary analysis in this report tested whether there was an overall association of AAO across all 22 SNPs. This was done by combining the association *p* values for each SNP using Brown's method.^21^ Essentially, this is Fisher's method for combining *p* values corrected for linkage disequilibrium between SNPs. The primary analysis used one‐sided *p* values for association in the same direction as that observed in GeM‐HD. In order to assess the overall directionality of the associations, we compared the significance to that obtained from a similar analysis using two‐sided *p* values. The analyses were performed on eight disease groups: all polyglutamine diseases (HD+SCAs), HD, all SCAs, SCA1, SCA2, SCA3, SCA6, and SCA7. *p* values were Bonferroni corrected for eight tests—this is conservative given that the disease groups are not independent. Individual SNPs significantly associated with AAO in each disease group were also noted. Because of small sample size, SCA17 was not analyzed independently, but was included in the analyses of all SCAs and HD+SCAs.

## Results

In the primary analysis, which tested the overall effect of all 22 SNPs on AAO, significant associations (after Bonferroni correction for eight tests) were observed for HD+SCAs (*p* = 1.43 × 10^–5^), HD (*p* = 0.00194), all SCAs (*p* = 0.00107), SCA2 (*p* = 0.00350), and SCA6 (*p* = 0.00162). The increased significance of these associations compared to an undirected test using two‐sided SNP *p* values (see supplementary Table 3) indicates concordance in the direction of effects across SNPs between these samples and GeM‐HD.^6^ In particular, the observed association with HD is a convincing replication of the GeM‐HD results in an independent sample.

As a secondary analysis, individual SNP associations were examined. Three of these were significant after Bonferroni correction for eight disease combinations and 22 SNPs (Table 4 and Supplementary Table 4): rs3512 in *FAN1* with all SCAs and HD+SCAs and rs1805323 in *PMS2* with HD+SCAs. Each association was in the same direction as in GeM‐HD.^6^ We did not replicate the most significant signal in GeM‐HD, rs146353869 (*p* = 4.30 × 10^–20^, associated with 6 years earlier age at motor onset of HD). This is likely due to our sample being much smaller than GeM‐HD and thus less well powered to find associations with SNPs with relatively low‐frequency minor allele frequency (MAF) such as rs146353869 (MAF = 0.017). However, rs3512, the most significant individual SNP in this study, indexes the second significant chromosome 15 signal in GeM‐HD (*p* = 5.28 × 10^–13^, associated with 1.4 years later onset of HD), and is in the 3′UTR (untranslated region) of *FAN1*. Three SNPs (rs1037700, rs5893603, and rs16869352) were found to be in high LD (*r* ^2^ > 0.8) in our sample with more significant SNPs from GeM‐HD. Removing these SNPs reduced the significance of the multi‐SNP associations with SCA2 and SCA6, although these remained nominally significant (see Supplementary Table 3). Finally (see Supplementary Table 3), all the significant multi‐SNP associations from the primary analysis remained significant after removing the most significant single SNP (rs3512), suggesting that the signal enrichment is not being driven by a single SNP.

**Table 4:** Individual SNPs Showing Significant Associations With the Age at Onset in Polyglutamine Diseases

SNP	Gene	Disease Group	Two‐Sided *p* Value	Same Direction as GeM‐HD?
**Significant After Bonferroni for 22 SNPs and 8 Disease Groupings (*p* < 2.84 × 10^–4^)**				
rs3512	*FAN1*	All (HD+SCAs)	1.52 × 10^–5^	Yes
rs1805323	*PMS2*	All (HD+SCAs)	3.14 × 10^–5^	Yes
rs3512	*FAN1*	All SCAs	2.22 × 10^–4^	Yes
**Significant After Bonferroni for 22 SNPs (*p* < 2.27 × 10^–3^)**				
rs1805323	*PMS2*	HD	3.14 × 10^–4^	Yes
rs1805323	*PMS2*	SCA1	1.67 × 10^–3^	Yes
rs1037699	*RRM2B*	SCA6	4.86 × 10^–4^	Yes
rs1037700	*RRM2B*	SCA6	5.47 × 10^–4^	Yes
rs5893603	*RRM2B*	SCA6	2.13 × 10^–3^	Yes

HD = Huntington's disease; SCA = spinocerebellar ataxia.

To visualize the combined effect of our SNPs on residual AAO a polygenic “age at onset score” was derived, defined as the sum of the number of minor alleles at each locus weighted by their effect size in GeM‐HD (note that negative scores here correspond to earlier AAO). The residual AAO for each quartile of this risk score was plotted in Figure 2. As expected, there was a positive correlation between residual AAO in our data and increasing age at onset score, although the effect was small—the score accounts for approximately 1% of the variance of residual AAO.

**Figure 1:**
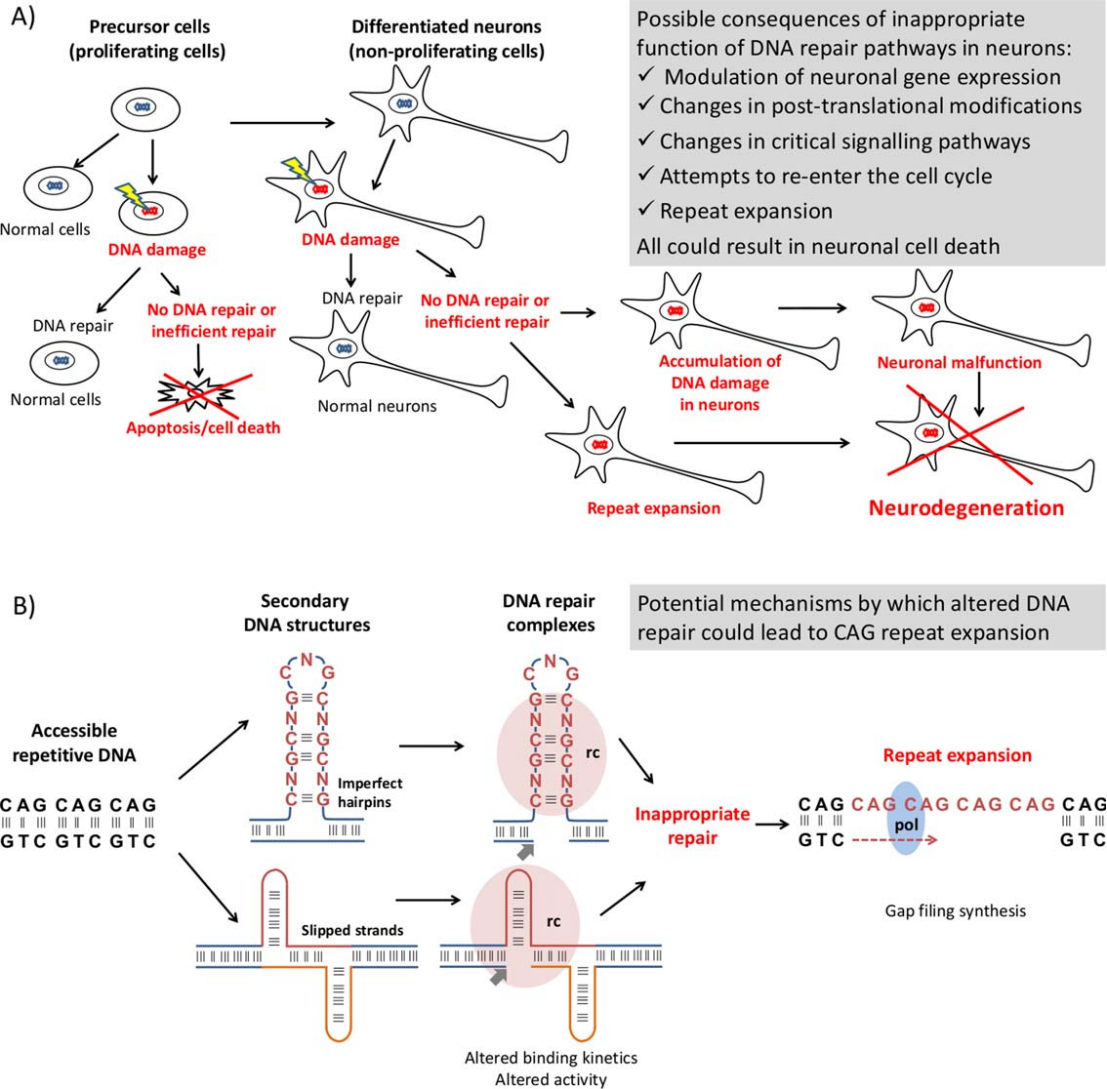
**Potential mechanisms through which variants in DNA repair genes identified in this study might lead to pathogenesis in polyglutamine diseases. (A) Overview of possible consequences of inappropriate function of DNA repair pathways in neurons. (B) Potential somatic expansion mechanism of the CAG repeats in polyglutamine diseases attributed to variation in genes encoding DNA repair proteins. The accessibility of repetitive DNA sequences during replication, transcription, etc., allows the formation of secondary DNA structures: SNPs in genes encoding DNA repair proteins may alter the kinetics or activity of DNA repair complexes (rc bobble). After endonuclease activity on the opposite strand (nick indicated by the thick arrow below), such impaired repair may lead to further expansion of the repeat tracts by consequent gap‐filling synthesis by DNA polymerase (pol bobble). SNPs 5 single‐nucleotide polymorphisms**. [Color figure can be viewed in the online issue, which is available at www.annalsofneurology.org.]

## Discussion

Our data implicate a common mechanism by which genetic variation in DNA repair pathways underlies age at onset of disease in multiple polyglutamine diseases. Alterations in DNA repair pathways could predispose to earlier onset by interacting with polyglutamine etiology at various levels. Rare loss‐of‐function variants in DNA repair genes cause multiple recessive ataxias^22^; *ATM* encodes a master regulator of DNA repair following double‐strand breaks,^23^ *PNPK* encodes a DNA‐specific kinase that facilitates DNA repair,^24^ *APTX* encodes a protein that interacts with PARP1 to mediate single strand DNA breaks,^25^ and mutations in *TDP1* also give defects in single‐strand break repair.^26^ The mechanisms by which neurodegeneration and ataxia result from these losses of function are not conclusively established, but there is substantial evidence for the fine control exercised by *ATM* being critical in cell division and cell death pathways, which could lead to neuronal loss.^27^ However, it is notable that none of the genes associated with recessive ataxia syndromes were identified to contain HD‐related variants in HD‐GeM.^6^

Repetitive DNA sequences can form unusual secondary structures^28^ to which DNA mismatch repair proteins bind and, in the process of repair, cause somatic instability (often expansion) of the CAG repeats. A number of enzymes with the ability to nick DNA and therefore necessitate DNA repair are known to promote CAG expansion and both somatic expansion and HD‐related phenotypes are ameliorated in mouse models by manipulating genes associated with DNA repair.^15^, ^29^, ^30^, ^31^, ^32^ Critically, delay in phenotype onset in HD mice was recently demonstrated through suppressing somatic expansion by crossing HTT knock in mice with *Ogg1^–/–^* mice, lacking the DNA cleaving 7,8‐dihydro‐8‐oxo‐guanine (8‐oxo‐G) glycosylase.^16^ Notably, the single most significant SNP in the present study, rs3512, is in the 3′UTR of *FAN1*, which has DNA endo/exonuclease activity. Larger CAG repeats are associated with more‐severe pathology and earlier disease onset in affected patients; therefore, somatic expansion provides a plausible mechanism by which the genetic variation we identify here could alter AAO of polyglutamine diseases (Fig 1). Additional consequences of impaired DNA repair cannot be discarded though (Fig 1A), and these may also be implicated in a wider range of neurodegenerative diseases, including several ataxic syndromes.^33^, ^34^

**Figure 2:**
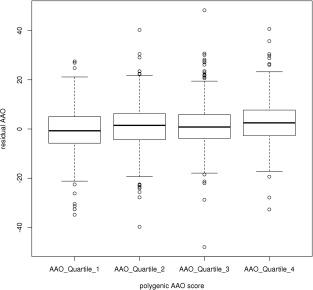
**Boxplot of residual AAO (across all samples) by quartiles of polygenic age at onset score. Polygenic score calculated by summing the number of minor alleles (weighted by their effect on age at onset in the GeM‐HD GWAS) across the 22 SNPs. Note that lower scores correspond to earlier‐than‐expected AAO and thus smaller residuals. AAO = age at onset; GWAS = genome‐wide association studies; SNPs = single‐nucleotide polymorphisms**.

There are several issues likely to have reduced the power of our study. The sample sizes for many of the SCAs were relatively small, and despite modeling the relationship of age of onset to CAG length separately for each disease, there is likely to be heterogeneity between diseases in this and potentially other respects that we have not been able to consider. We could not account for interruptions of pure CAG repeat tracts, which may stabilize repeat instability^35^; thus, our power to detect any effects mediated by somatic instability may have been reduced. Nevertheless, we have shown that DNA repair genes as a group significantly modify AAO in the polyglutamine diseases taken together, in HD, in all SCAs, SCA2, and SCA6. Additionally, we have identified potential modifier SNPs in HD, SCA1, and SCA6 (Table 4 and Supplementary Table 4). The effects of these SNPs on AAO are quite small, and it would be worth repeating the analysis with larger samples and more SNPs as the predictive power of such polygenic risk scores increases as sample size and number of variants genotyped increase.^36^, ^37^, ^38^

By suggesting common mechanisms for polyglutamine diseases, our findings offer novel therapeutic opportunities in multiple diseases along with the potential to improve clinical trial design by stratifying subject variability. Molecules targeting DNA repair have been developed and are used in the clinic to treat cancers,^39^, ^40^ and such therapeutics, along with others in development, may prove useful in some or all of the polyglutamine diseases. Furthermore, these shared mechanisms may extend to diseases associated with non‐CAG and nontranslated repeats, most likely in those that show somatic instability.

## Author Contributions

M.S., H.H., S.J.T., and L.J. were responsible for concept and study design. C.B., D.H.M., M.F., S.W., A.B., C.G., G.S. G.Ko., G.Ka., M.P., P.Y.‐G., L.E.G.‐V., M.E.A.‐V., M.L., M.R., B.T., M.S., N.W., P.G., the SPATAX Network, A.R., P.H., H.H., S.J.T., and L.J. were responsible for data acquisition and analysis. C.B., D.H.M., M.F., S.W., P.H., H.H., S.J.T., and L.J. were responsible for drafting manuscript and figures. C.B., D.H.M., M.F., and S.W. contributed equally to this work. P.H., H.H., S.J.T., and L.J. supervised this work.

## Potential Conflicts of Interest

The author declare no conflicts of interest.

## Supporting information

Additional supporting information can be found in the online version of this article.

Supporting Information

## References

[1] McMurray CT . Mechanisms of trinucleotide repeat instability during human development. Nat Rev Genet 2010;11:786–799. 20953213 10.1038/nrg2828 PMC3175376

[2] Gatchel JR , Zoghbi HY . Diseases of unstable repeat expansion: mechanisms and common principles. Nat Rev Genet 2005;6:743–755. 16205714 10.1038/nrg1691

[3] Tezenas du Montcel S , Durr A , Bauer P , et al. Modulation of the age at onset in spinocerebellar ataxia by CAG tracts in various genes. Brain 2014;137(pt 9):2444–2455. 24972706 10.1093/brain/awu174 PMC4132646

[4] Wexler NS , Lorimer J , Porter J , et al.; U.S.‐Venezuela Collaborative Research Project . Venezuelan kindreds reveal that genetic and environmental factors modulate Huntington's disease age of onset. Proc Natl Acad Sci U S A 2004;101:3498–3503. 14993615 10.1073/pnas.0308679101 PMC373491

[5] van de Warrenburg BP , Hendriks H , Durr A , et al. Age at onset variance analysis in spinocerebellar ataxias: a study in a Dutch‐French cohort. Ann Neurol 2005;57:505–512. 15747371 10.1002/ana.20424

[6] Genetic Modifiers of Huntington's Disease (GeM‐HD) Consortium . Identification of genetic factors that modify clinical onset of Huntington's disease. Cell 2015;162:516–526. 26232222 10.1016/j.cell.2015.07.003 PMC4524551

[7] Bettencourt C , Raposo M , Kazachkova N , et al. The APOE epsilon2 allele increases the risk of earlier age at onset in Machado‐Joseph disease. Arch Neurol 2011;68:1580–1583. 22159055 10.1001/archneurol.2011.636

[8] Raposo M , Ramos A , Bettencourt C , Lima M . Replicating studies of genetic modifiers in spinocerebellar ataxia type 3: can homogeneous cohorts aid? Brain 2015;138(pt 2):e389. 26173860 10.1093/brain/awv206

[9] Peng H , Wang C , Chen Z , et al. APOE epsilon2 allele may decrease the age at onset in patients with spinocerebellar ataxia type 3 or Machado‐Joseph disease from the Chinese Han population. Neurobiol Aging 2014;35:2179.e15–e18. 24746364 10.1016/j.neurobiolaging.2014.03.020

[10] Lopez Castel A , Cleary JD , Pearson CE . Repeat instability as the basis for human diseases and as a potential target for therapy. Nat Rev Mol Cell Biol 2010;11:165–170. 20177394 10.1038/nrm2854

[11] Iyer RR , Pluciennik A , Napierala M , Wells RD . DNA triplet repeat expansion and mismatch repair. Annu Rev Biochem 2015;84:199–226. 25580529 10.1146/annurev-biochem-060614-034010 PMC4845744

[12] Gomes‐Pereira M , Monckton DG . Chemical modifiers of unstable expanded simple sequence repeats: what goes up, could come down. Mutat Res 2006;598:15–34. 16500684 10.1016/j.mrfmmm.2006.01.011

[13] Gonitel R , Moffitt H , Sathasivam K , et al. DNA instability in postmitotic neurons. Proc Natl Acad Sci U S A 2008;105:3467–3472. 18299573 10.1073/pnas.0800048105 PMC2265187

[14] Swami M , Hendricks AE , Gillis T , et al. Somatic expansion of the Huntington's disease CAG repeat in the brain is associated with an earlier age of disease onset. Hum Mol Genet 2009;18:3039–3047. 19465745 10.1093/hmg/ddp242 PMC2714728

[15] Dragileva E , Hendricks A , Teed A , et al. Intergenerational and striatal CAG repeat instability in Huntington's disease knock‐in mice involve different DNA repair genes. Neurobiol Dis 2009;33:37–47. 18930147 10.1016/j.nbd.2008.09.014 PMC2811282

[16] Budworth H , Harris FR , Williams P , et al. Suppression of somatic expansion delays the onset of pathophysiology in a mouse model of Huntington's disease. PLoS Genet 2015;11:e1005267. 26247199 10.1371/journal.pgen.1005267 PMC4527696

[17] Mason AG , Tome S , Simard JP , et al. Expression levels of DNA replication and repair genes predict regional somatic repeat instability in the brain but are not altered by polyglutamine disease protein expression or age. Hum Mol Genet 2014;23:1606–1618. 24191263 10.1093/hmg/ddt551 PMC3929096

[18] Pearson CE , Nichol Edamura K , Cleary JD . Repeat instability: mechanisms of dynamic mutations. Nat Rev Genet 2005;6:729–742. 16205713 10.1038/nrg1689

[19] Tabrizi SJ , Scahill RI , Owen G , et al. Predictors of phenotypic progression and disease onset in premanifest and early‐stage Huntington's disease in the TRACK‐HD study: analysis of 36‐month observational data. Lancet Neurol 2013;12:637–649. 23664844 10.1016/S1474-4422(13)70088-7

[20] Purcell S , Neale B , Todd‐Brown K , et al. PLINK: a tool set for whole‐genome association and population‐based linkage analyses. Am J Hum Genet 2007;81:559–575. 17701901 10.1086/519795 PMC1950838

[21] Brown MB . A method for combining non‐independent, one‐sided tests of significance. Biometrics 1975;31:987–992.

[22] Storey E . Genetic cerebellar ataxias. Semin Neurol 2014;34:280–292. 25192506 10.1055/s-0034-1386766

[23] Ambrose M , Gatti RA . Pathogenesis of ataxia‐telangiectasia: the next generation of ATM functions. Blood 2013;121:4036–4045. 23440242 10.1182/blood-2012-09-456897 PMC3709651

[24] Bras J , Alonso I , Barbot C , et al. Mutations in PNKP cause recessive ataxia with oculomotor apraxia type 4. Am J Hum Genet 2015;96:474–479. 25728773 10.1016/j.ajhg.2015.01.005 PMC4375449

[25] Harris JL , Jakob B , Taucher‐Scholz G , et al. Aprataxin, poly‐ADP ribose polymerase 1 (PARP‐1) and apurinic endonuclease 1 (APE1) function together to protect the genome against oxidative damage. Hum Mol Genet 2009;18:4102–4117. 19643912 10.1093/hmg/ddp359

[26] Takashima H , Boerkoel CF , John J , et al. Mutation of TDP1, encoding a topoisomerase I‐dependent DNA damage repair enzyme, in spinocerebellar ataxia with axonal neuropathy. Nat Genet 2002;32:267–272. 12244316 10.1038/ng987

[27] Shiloh Y , Ziv Y . The ATM protein kinase: regulating the cellular response to genotoxic stress, and more. Nat Rev Mol Cell Biol 2013;14:197–210. 23847781

[28] Mirkin SM . Expandable DNA repeats and human disease. Nature 2007;447:932–940. 17581576 10.1038/nature05977

[29] Wheeler VC , Lebel LA , Vrbanac V , et al. Mismatch repair gene Msh2 modifies the timing of early disease in Hdh(Q111) striatum. Hum Mol Genet 2003;12:273–281. 12554681 10.1093/hmg/ddg056

[30] Tome S , Manley K , Simard JP , et al. MSH3 polymorphisms and protein levels affect CAG repeat instability in Huntington's disease mice. PLoS Genet 2013;9:e1003280. 23468640 10.1371/journal.pgen.1003280 PMC3585117

[31] Kovtun IV , Liu Y , Bjoras M , et al. OGG1 initiates age‐dependent CAG trinucleotide expansion in somatic cells. Nature 2007;447:447–452. 17450122 10.1038/nature05778 PMC2681094

[32] Goula AV , Berquist BR , Wilson DM, III , et al. Stoichiometry of base excision repair proteins correlates with increased somatic CAG instability in striatum over cerebellum in Huntington's disease transgenic mice. PLoS Genet 2009;5:e1000749. 19997493 10.1371/journal.pgen.1000749 PMC2778875

[33] Barzilai A , Biton S , Shiloh Y . The role of the DNA damage response in neuronal development, organization and maintenance. DNA Repair 2008;7:1010–1027. 18458000 10.1016/j.dnarep.2008.03.005

[34] Madabhushi R , Pan L , Tsai LH . DNA damage and its links to neurodegeneration. Neuron 2014;83:266–282. 25033177 10.1016/j.neuron.2014.06.034 PMC5564444

[35] Menon RP , Nethisinghe S , Faggiano S , et al. The role of interruptions in polyQ in the pathology of SCA1. PLoS Genet 2013;9:e1003648. 23935513 10.1371/journal.pgen.1003648 PMC3723530

[36] Dudbridge F . Power and predictive accuracy of polygenic risk scores. PLoS Genet 2013;9:e1003348. 23555274 10.1371/journal.pgen.1003348 PMC3605113

[37] International Schizophrenia Consortium , Purcell SM , Wray NR , et al. Common polygenic variation contributes to risk of schizophrenia and bipolar disorder. Nature 2009;460:748–752. 19571811 10.1038/nature08185 PMC3912837

[38] Schizophrenia Working Group of the Psychiatric Genomics Consortium . Biological insights from 108 schizophrenia‐associated genetic loci. Nature 2014;511:421–427. 25056061 10.1038/nature13595 PMC4112379

[39] Farmer H , McCabe N , Lord CJ , et al. Targeting the DNA repair defect in BRCA mutant cells as a therapeutic strategy. Nature 2005;434:917–921. 15829967 10.1038/nature03445

[40] Jackson SE , Chester JD . Personalised cancer medicine. Int J Cancer 2015;137:262–266. 24789362 10.1002/ijc.28940

