# Supplementary material for: DNA repair pathways underlie a common genetic mechanism modulating onset in polyglutamine diseases
Source: Ann Neurol. 2016 May 6;79(6):983–90. doi: 10.1002/ana.24656 (PMC4914895; doi:10.1002/ana.24656)
Supplement: Supplementary file 1 — Supporting Information [file ANA-79-983-s001.docx]

Supplementary Materials

## Supplementary Table 1: Characteristics of single nucleotide polymorphisms (SNPs) used in this study

| **SNP ID** | **Chr:position (bp) (GRCh37/hg19)** | **Gene symbol** | **Functional annotation** | **P  (GeM-HD)** | **MAF*** | **Genotype call rate*** | **P  (HWE)*** |
| --- | --- | --- | --- | --- | --- | --- | --- |
| rs1800937 | 2:48025764 | *MSH6* | Stop_gained | 4.30E-03 | 0.074 | 0.973 | 0.840 |
| rs4150407 | 2:128049631 | *ERCC3* | Intron_variant | 4.60E-04 | 0.479 | 0.964 | 0.003 |
| rs5742933 | 2:190649316 | *PMS1* | NMD_transcript_variant | 9.49E-04 | 0.205 | 0.972 | 1.000 |
| rs1799977 | 3:37053568 | *MLH1* | Missense_variant | 7.16E-07 | 0.28 | 0.966 | 0.354 |
| rs6151792 | 5:80056961 | *MSH3* | Intron_variant | 2.09E-04 | 0.117 | 0.978 | 0.706 |
| rs115109737 | 5:80102444 | *MSH3* | Intron_variant | 4.50E-04 | 0.041 | 0.980 | 0.489 |
| rs71636247 | 5:80118976 | *MSH3* | Intron_variant | 2.55E-04 | 0.034 | 0.976 | 1.000 |
| rs1805323 | 7:6026942 | *PMS2* | Missense_variant | 3.04E-02 | 0.043 | 0.975 | 0.736 |
| rs12531179 | 7:6028687 | *PMS2* | Intron_variant | 3.84E-05 | 0.169 | 0.971 | 0.925 |
| rs3735721 | 8:103217695 | *RRM2B* | 3’_UTR_variant | 5.68E-07 | 0.083 | 0.971 | 0.058 |
| rs1037700 | 8:103250775 | *RRM2B* | Intron_variant | 5.03E-08 | 0.094 | 0.973 | 0.002 |
| rs5893603 | 8:103250839 | *RRM2B* | Frameshift_variant | 4.28E-08 | 0.093 | 0.973 | 0.007 |
| rs1037699 | 8:103250930 | *RRM2B* | Missense_variant | 2.70E-08 | 0.094 | 0.976 | 0.002 |
| rs16869352 | 8:103306033 | *UBR5* | Synonymous_variant | 4.01E-07 | 0.08 | 0.975 | 0.030 |
| rs61752302 | 8:103311153 | *UBR5* | Synonymous_variant | 3.03E-03 | 0.026 | 0.977 | 0.621 |
| rs72734283 | 14:75495059 | *MLH3* | Intron_variant | 4.32E-03 | 0.089 | 0.971 | 0.623 |
| rs175080 | 14:75513828 | *MLH3* | Missense_variant | 7.72E-03 | 0.435 | 0.971 | 0.447 |
| rs146353869 | 15:31126401 | *FAN1* | Intron_variant | 4.30E-20 | 0.017 | 0.973 | 1.000 |
| rs114136100 | 15:31197976 | *FAN1* | Synonymous_variant | 8.49E-16 | 0.019 | 0.976 | 0.423 |
| rs150393409 | 15:31202961 | *FAN1* | Missense_variant | 9.34E-18 | 0.013 | 0.975 | 1.000 |
| rs3512 | 15:31235005 | *FAN1* | 3’_UTR_variant | 5.28E-13 | 0.283 | 0.973 | 1.000 |
| rs20579 | 19:48668830 | *LIG1* | NMD_transcript_variant | 6.65E-03 | 0.134 | 0.942 | 0.732 |

SNPs were selected from the most significant genes (gene-wide p<0.1) in the “DNA repair pathway cluster” from the GeM-HD analysis[^1^](#_ENREF_1) (listed in Table S4 of GeM-HD). Genes annotated by the SNPs are indicated. *Refers to the current study. Chr = chromosome; MAF = minor allele frequency; HWE = Hardy–Weinberg equilibrium.

## Supplementary Table 2: Seed sense sequences for SNP KASP assay design

| **SNPs** | **HGVS Names** | **SNP to Chromosome** | **Seed sense sequences for KASP assay design** |
| --- | --- | --- | --- |
| rs1800937 | NC_000002.11:g.48025764C>T | Forward | TTGCCTGGCAGGTAGGCACAACTTA**[C>T]**GTAACAGATAAGAGTGAAGAAGATA |
| rs4150407 | NC_000002.11:g.128049631T>C | Reverse | AGTACACAATGGGAAGGTGGTCCAT**[A>G]**GACAAGAGCCTTCACCAGAAACTGA |
| rs5742933 | NC_000002.11:g.190649316G>C | Forward | GTAATTGCCTGCCTCGCGCTAGCAG**[G>C]**AAGGTAGTGTGGTGTGACTAACGGG |
| rs1799977 | NC_000003.11:g.37053568A>G | Forward | CTCAACCGTGGACAATATTCGCTCC**[A>G]**TCTTTGGAAATGCTGTTAGTCGGTA |
| rs6151792 | NC_000005.9:g.80056961C>T | Forward | TCACACAGCCATGTAAAATTAGGCC**[C>T]**GCAGACAATTCGAAGGAGGAGAAAA |
| rs115109737 | NC_000005.9:g.80102444G>A | Forward | GAATCACACAAGCTTATTTGCTATA**[G>A]**CATTATAATAACTTTTTACATCTGT |
| rs71636247 | NC_000005.9:g.80118976A>G | Forward | TGTATAAATATATGTGGAGAAAACC**[A>G]**TCTAGATAGAAGGCTTATTCCAAAA |
| rs1805323 | NC_000007.13:g.6026942G>T | Reverse | TCCAGTCACGGACCCAGTGACCCTA**[C>A]**GGACAGAGCGGAGGTGGAGAAGGAC |
| rs12531179 | NC_000007.13:g.6028687C>T | Forward | ATTTTTAGTAGAGACAGAGTTTCAC**[C>T]**GTGTTAGATAGTCTCGATCTCCTGA |
| rs3735721 | NC_000008.10:g.103217695A>G | Forward | GCTGGGGCCAGCTTAGTTGTAAGAA**[A>G]**AACTATTATTGTATATAATTGGACA |
| rs1037700 | NC_000008.10:g.103250775G>C | Reverse | GGCCTCAGGCCGGGGTGAGACTTAC**[C>G]**CCTGCGTTTATCCGCCTCACGCTCT |
| rs5893603 | NC_000008.10:g.103250839_103250840insG | Forward | TTGGCTGGCCCCGGGGCAGAGCAGC**[->G]**GAGCGGGACGCAAACCCAAAGTCAG |
| rs1037699 | NC_000008.10:g.103250930C>T | Reverse | AGGACAGGCCTGTCCGCCCGCCCTC**[G>A]**CCGCAGCCTGGCTTCGTCGTTGCGA |
| rs16869352 | NC_000008.10:g.103306033T>C | Forward | CAGCGTAAGGTAGCAATGCTTGGAA**[T>C]**ACACGCTTGCATTTTCCAATTGGCT |
| rs61752302 | NC_000008.10:g.103311153C>T | Forward | ACAATTTCAATATAAAATGAGCATT**[C>T]**GCCTTTCGATCCTTGGATTCTACTA |
| rs72734283 | NC_000014.8:g.75495059A>G | Forward | ATTATTTTATGATTTGACCTTGACA**[A>G]**CCCATCTAGCCAACTCCCATCCAGT |
| rs175080 | NC_000014.8:g.75513828G>A | Forward | GGTCATAGGACTTTCTCTCAAACTA**[G>A]**GCATCTGTTGTTCTAAACAATCTTC |
| rs146353869 | NC_000015.9:g.31126401C>A | Forward | AATGGTATGTATTAAAATGTGAATC**[C>A]**CAAGAGTGATGTGTCACTGTGCACT |
| rs114136100 | NC_000015.9:g.31197976C>T | Forward | GCTGCAATGGTCCTGGTCAAACAAC**[C>T]**GGTCATCCTTACTACCTTCGGAGTT |
| rs150393409 | NC_000015.9:g.31202961G>A | Forward | GCCTTTCTCAAATTGGCCAAACAGC**[G>A]**TTCAGTCTGCACTTGGGGCAAGAAT |
| rs3512 | NC_000015.9:g.31235005G>C | Reverse | ACAGAGAGCGTTAAAAGTAAAGGCA**[C>G]**TTCCAAGAGTAACACTGCTAATGCG |
| rs20579 | NC_000019.9:g.48668830G>A | Reverse | GCTGGACAGGAAGGGAGAATTCTGA**[C>T]**GCCAACATGCAGCGAAGTATCATGT |

Note that genotypes for SNPs in reverse orientation to chromosome given by our KASP assays (highlighted in red) are complementary (reverse) to HGVS nomenclature.

## Supplementary Table 3: Results of combined analysis of SNPs

| **Disease Group** | **GeM-HD concordance?** | **P (All SNPs)** | **P (High LD SNPs removed)** | **P (rs3512 removed)** |
| --- | --- | --- | --- | --- |
| ALL (HD+SCAs) | non directional | 4.74x10^-4^ * | 2.26x10^-4^ * | 0.00492 * |
|  | Same as GeM-HD | 1.43x10^-5^ * | 6.98x10^-6^ * | 2.26x10^-4^ * |
| HD | non directional | 0.0226 | 0.00775 | 0.0364 |
|  | Same as GeM-HD | 0.00194 * | 4.63x10^-4^ * | 0.00394 * |
| SCAs | non directional | 0.0188 | 0.0236 | 0.0771 |
|  | Same as GeM-HD | 0.00107 * | 0.00142 * | 0.00667 * |
| SCA1 | non directional | 0.376 | 0.386 | 0.444 |
|  | Same as GeM-HD | 0.416 | 0.287 | 0.524 |
| SCA2 | non directional | 0.0230 | 0.0629 | 0.0233 |
|  | Same as GeM-HD | 0.00350 * | 0.0138 | 0.00442 * |
| SCA3 | non directional | 0.176 | 0.114 | 0.355 |
|  | Same as GeM-HD | 0.0809 | 0.0381 | 0.205 |
| SCA6 | non directional | 0.00588 * | 0.0735 | 0.00506 * |
|  | Same as GeM-HD | 0.00162 * | 0.0340 | 0.00163 * |
| SCA7 | non directional | 0.155 | 0.217 | 0.297 |
|  | Same as GeM-HD | 0.0447 | 0.0885 | 0.113 |

P-values in this table obtained by combining single-SNP p-values using Brown’s method[^2^](#_ENREF_2), allowing for LD between SNPs. Non-directional analysis combines two-sided p-values. “Same as GeM-HD” analyses combine one-sided p-values in the same direction as the SNP effects observed in GeM-HD study^6^. In the “High LD SNPs removed” analysis, rs1037700, rs5893603 and rs16869352 were removed due to high LD (r2>0.8) with more significant SNPs in GeM-HD. * P-values that satisfy Bonferroni correction for 8 disease group tests. Note that SCA17 was included in the “HD+SCAs” and “All SCAs” grouped analyses, but was not tested independently due to small sample size. HD – Huntington’s disease; SCA – spinocerebellar ataxia

Supplementary Table 4: Single SNP associations

Beta denotes the effect size – that is, the number of years added to or subtracted from the expected age at onset for each copy of the minor allele (A1). MAF denotes the frequency of the minor allele in GeM-HD[**^1^**](#_ENREF_1) (Column 6) and the present study (Column 11). P values highlighted bold and “*” satisfy Bonferroni correction for 22 SNPs; those highlighted bold and “**” satisfy Bonferroni correction for 8 disease groups and 22 SNPs. Note that for SNPs in reverse orientation to chromosome (rs4150407, rs1805323, rs1037700, rs1037699, rs3512, and rs20579) genotypes given by KASP assays (current study) are complementary to those obtained in GeM-HD, which uses HGVS nomenclature (see Supplementary Table 1), corresponding to the same allele.

# Supplementary References

1. Genetic Modifiers of Huntington’s Disease (GeM-HD) Consortium. Identification of Genetic Factors that Modify Clinical Onset of Huntington's Disease. Cell. 2015 Jul 30;162(3):516-26.

2. Brown MB. A method for combining non-independent, one-sided tests of significance. Biometrics. 1975:987-92.
